# Necrotising Fasciitis During the COVID-19 Pandemic: An Australian Hospital Network Experience

**DOI:** 10.1007/s00268-023-07040-2

**Published:** 2023-05-03

**Authors:** Quoc Dung Nguyen, Jason Diab, David Khaicy, Vanessa Diab, Zachias Hopkins, Lai Heng Foong, Christophe R. Berney

**Affiliations:** 1 Department of Emergency Medicine, Bankstown-Lidcombe Hospital, Eldridge Road, Bankstown, NSW Australia; 2 School of Medicine, University of Notre Dame, Sydney, Australia; 3 School of Medicine, University of New South Wales, Sydney, Australia

## Abstract

**Background:**

The clinical presentations of diseases and the provision of global healthcare services have been negatively affected by the COVID-19 pandemic. Our study aimed to determine the impact of this global pandemic on presentations of necrotising fasciitis (NF).

**Methods:**

A retrospective study was conducted of adult patients with NF in South West Sydney Local Health District from January 2017 to October 2022. An analysis of sociodemographic and clinical outcomes was performed comparing the COVID-19 cohort (2020–2022) and the pre-COVID-19 cohort (2017–2019).

**Results:**

Sixty-five patients were allocated to the COVID-19 cohort, and 81 patients were in the control cohort. The presentation to hospitals of the COVID-19 cohort was significantly delayed compared to the control cohort (6.1 vs. 3.2 days, *P* < 0.001). Patients of the age group of 40 years and younger experienced prolonged operative time (1.8 vs. 1.0 h, *P* = 0.040), higher number of operations (4.8 vs. 2.1, *P* = 0.008), and longer total length of stay (LoS) (31.3 vs. 10.3 days, *P* = 0.035) during the pandemic. The biochemical, clinical, or post-operative outcomes of two groups were not significantly different.

**Conclusion:**

This multi-centre study showed that the COVID-19 pandemic delayed presentations of NF but did not result in any significant overall changes in operative time, ICU admissions, LoS, and mortality rate. Patients aged less than 40 years in the COVID-19 group were likely to experience prolonged operative time, higher number of operations, and greater LoS.

## Introduction

On 11th March 2020, the World Health Organisation declared the novel coronavirus-2 (SARS-CoV-2) outbreak a global pandemic [1]. 5 day later, “social distancing” was introduced by the Australian government, and significant changes in the public health system, including suspension of non-essential surgical procedures, were gradually implemented [2]. The nature of diseases presentations and the provision of health services have been largely affected since the pandemic. The reduction in clinical and emergency presentations has shown to influence the rate of hospitalisation and changes in patterns of admissions [3–7].

We previously recognised an unusual pattern of delayed presentations of patients with necrotising fasciitis (NF) at a Sydney metropolitan hospital [8], most probably because of concern of contracting SARS-CoV-2 [9, 10]. NF is a rare bacterial infection of soft tissue and fascia, which can spread rapidly in the multiple soft tissue layers and may result in potentially fatal outcomes [4]. In the community, the incidence of this life-threatening soft tissue infection ranges from 0.3 to 15 per 100,000 [11, 12] with a mortality of around 20.6%, it is considered a surgical emergency requiring prompt diagnosis and treatment to prevent its aggressive and rapidly progressive nature [13].

This retrospective study aims to examine the effect of the COVID-19 pandemic on presentation and clinical outcomes for NF in South-Western Sydney.

## Methods

### Study design and data collection

All cases of NF, which were documented at four major hospitals in South-Western Sydney Local Health District (SWSLHD)^1^ from January 2017 to October 2022, were retrospectively reviewed. Patients with a confirmed diagnosis of NF, who aged 16 years old and above, were included. They were categorised into the COVID-19 cohort, including patients who presented to hospital from January 2020 to the end of October 2022, and the pre-COVID-19 (control) cohort, including those who presented from 2017 to 2019. Further subgroup analysis was similarly performed during the two lockdown periods, which were March–July 2020 and August–October 2021. Data was retrospectively collected through electronic medical records and theatre records. The collected parameters were social demography, clinical presentation, past medical history, Charlson comorbidity index [11], biochemical data, Laboratory Risk Indicator for Necrotizing Fasciitis (LRINEC) score [14], time from presentation to theatre, duration of operation, surgical procedure, number of surgeries, post-operative complications, length of stay (LoS), and mortality (in-hospital, 30-day, 90-day, and 1-year).

### Statistical analysis

Data were presented as mean ± standard deviation (SD) for continuous variables or frequencies (n) and percentages for categorical variables. The statistical software SPSS (Version 27.0) was used for performing statistical analyses. Fisher’s exact test was used for analysing categorical data, and continuous variables were examined by using Mann Whitney U test. Two-sided *P* values < 0.05 were considered statistically significant.

## Results

### Demographics

**Fig. 1:**
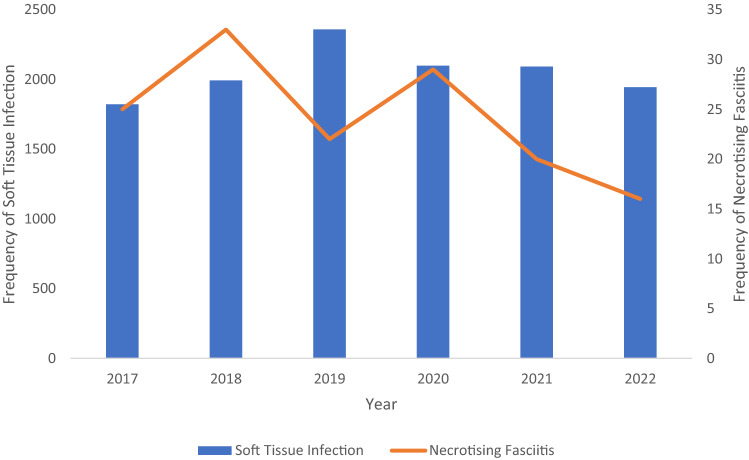
Number of soft tissue infection and necrotising fasciitis cases from January 2017 to October 2022

There were 11,449 emergency operations for soft tissue infections from January 2017 to October 2022 in SWSLHD with 146 patients who had NF (1.3%). The percentage of NF to soft tissue infection fluctuated between 0.8% and 1.7% from January 2017 to October 2022 with a decrease in the number of NF cases between pre-pandemic and pandemic years (*P* = 0.214) (Fig. 1). During the pre-pandemic period, there were 81 cases compared to 65 cases (*P* = 0.070, Table 1).

**Table 1:** Comparative analysis between the COVID-19 cohort and the control cohort

	Non COVID-19 (2017–2019)	COVID-19 (2020–2022)	*P* value
Number of cases	81	65	
Average cases per month (SD)	2.2 (± 1.2)	1.8 (± 1.8)	0.070
*Demographic Information*			
Age [years], mean (SD)	55.5 (± 18.3)	57.4 (± 18.8)	0.529
Age 40 and below, n (%)	15 (18.5%)	17 (26.2%)	0.289
Age 41–64, n (%)	40 (49.4%)	24 (36.9%)	
Age 65 and above, n (%)	26 (32.1%)	24 (36.9%)	
Female, n (%)	33 (40.7%)	14 (21.5%)	0.020*
Height [cm], mean (SD)	168.9 (± 9.0)	171.8 (± 10.5)	0.085
Weight [kg], mean (SD)	94.1 (± 30.1)	106.2 (± 43.3)	0.059
Body mass index (BMI) [kg/m^2^], mean (SD)	32.6 (± 9.5)	35.0 (± 12.4)	0.223
Obesity	37 (52.9%)	32 (57.1%)	0.719
CALD, n (%)	31 (38.3%)	24 (36.9%)	1.000
Smoker	24 (29.6%)	23 (20.9%)	0.481
*Clinical Presentation and Symptomology*			
Onset of symptoms till presentation to the Emergency Department [days], mean (SD)	3.2 (± 2.0)	6.1 (± 4.7)	<0.001*
Age 40 and below	2.6 (± 1.9)	5.9 (± 3.1)	0.001*
Age 41–64	3.4 (± 1.9)	6.4 (± 6.1)	0.005*
Age 65 and above	3.4 (± 2.1)	5.8 (± 4.1)	0.010*
Clinical signs, n (%)			
Hypoaesthesia	6 (7.4%)	6 (9.2%)	0.767
Disproportionate pain at the site of injury	79 (97.5%)	63 (96.9%)	1.000
Swelling of the skin	78 (96.3%)	60 (92.3%)	0.467
Erythema of the skin	76 (93.8%)	60 (92.3%)	0.752
Skin crepitus	0 (0.0%)	3 (4.6%)	0.086
Haemorrhagic bullae or blisters	5 (6.2%)	7 (10.8%)	0.372
Symptoms, n (%)			
Presence of abdominal pain	9 (11.1%)	7 (10.8%)	1.000
Site of Injury			
Neck	3 (3.7%)	0 (0.0%)	0.254
Chest/Breast	4 (4.9%)	0 (0.0%)	0.129
Upper limb	9 (11.1%)	6 (9.2%)	0.789
Abdomen	7 (8.6%)	5 (7.7%)	1.000
Back	3 (3.7%)	2 (3.1%)	1.000
Groin	5 (6.2%)	7 (10.8%)	0.372
Buttock	7 (8.6%)	2 (3.1%)	0.299
Perineum	26 (32.1%)	28 (43.1%)	0.227
Lower limb	17 (21.0%)	15 (23.1%)	0.841
Previous trauma to the site	9 (11.1%)	5 (7.7%)	0.579
Influenza like symptoms	8 (9.9%)	5 (7.7%)	0.774
Decreased urine output	1 (1.2%)	8 (12.3%)	0.011*
SIRS, n (%)	37 (45.7%)	35 (53.8%)	0.405
Sepsis, n (%)	11 (13.6%)	14 (21.5%)	0.269
qSOFA Score, mean (SD)	0.7 (± 0.8)	0.6 (± 0.8)	0.480
*Medical Comorbidities*			
Pre-existing medical illness, n (%)	47 (58.0%)	48 (42.3%)	0.055
Type 1 diabetes mellitus	1 (1.2%)	1 (1.5%)	1.000
Type 2 diabetes mellitus	31 (32.7%)	28 (43.1%)	0.612
Previous abdominal surgery, n (%)	15 (18.5%)	12 (18.5%)	1.000
Charlson comorbidity index, mean (SD)	2.9 (± 2.5)	2.8 (± 2.5)	0.949
Charlson score 0, n (%)	19 (23.5%)	11 (16.9%)	0.411
Charlson score 1, n (%)	10 (12.3%)	13 (20.0%)	0.255
Charlson score 2, n (%)	9 (11.1%)	9 (13.8%)	0.623
Charlson score ≥ 3, n (%)	43 (53.1%)	31 (47.7%)	0.618
*Biochemical parameters [normal range], mean (SD)*			
White cell count (× 10^9^/L) [4.0–10.0]	17.9 (± 10.7)	17.0 (± 7.2)	0.567
Neutrophil (× 10^9^/L) [2.0–7.0]	14.5 (± 7.8)	14.2 (± 6.8)	0.845
C reactive protein (mg/L) [<4.9]	245.9 (± 147.2)	249.8 (± 147.9)	0.876
Haemoglobin (g/L) [130–170]	127.6 (± 24.7)	127.0 (± 28.1)	0.886
Platelets (× 10^9^/L) [150–400]	271.5 (± 146.7)	257.7 (± 118.3)	0.542
Creatinine (µmol/L) [60–110]	142.0 (± 131.6)	163.4 (± 150.6)	0.360
Urea (mmol/L) [4.0–9.0]	10.4 (± 8.9)	12.0 (± 9.6)	0.315
Albumin (g/L) [33–48]	28.5 (± 6.4)	26.4 (± 7.6)	0.077
Lactate (mmol/L) [<1.9]	2.9 (± 2.4)	2.6 (± 2.4)	0.539
Sodium (mmol/L) [135—145]	133.5 (± 5.5)	133.4 (± 6.1)	0.958
Glucose (mmol/L) [7.8–11.0]	11.8 (± 8.3)	13.4 (± 11.5)	0.334
LRINEC category, mean (SD)	6.5 (3.2)	6.4 (3.4)	0.740
Low risk (≤5), n (%)	29 (28.8%)	23 (35.4%)	1.000
Moderate risk (6–7), n (%)	16 (19.8%)	13 (20.0%)	1.000
High risk (≥8), n (%)	36 (44.4%)	29 (44.6%)	1.000
*Imaging*			
CT scan, n (%)	44 (54.3%)	32 (49.2%)	0.618
*Management*			
ASA status, n (%)			
ASA 1	4 (4.9%)	2 (3.1%)	0.693
ASA 2	11 (13.6%)	10 (15.4%)	0.815
ASA 3	44 (54.3%)	29 (44.6%)	0.318
ASA 4	17 (21.0%)	24 (36.9%)	0.042*
ASA 5	5 (6.2%)	0 (0.0%)	0.066
Time to theatre [hours], mean (SD)	10.9 (± 11.0)	13.4 (± 11.5)	0.946
Age 40 and below	11.8 (± 9.9)	8.4 (± 7.1)	0.277
Age 41–64	9.4 (± 12.8)	9.6 (± 6.4)	0.950
Age 65 and above	12.8 (± 8.3)	13.7 (± 10.5)	0.723
Operative time [hours], mean (SD)	1.3 (± 1.0)	1.3 (± 1.0)	0.950
Age 40 and below	1.0 (± 0.7)	1.8 (± 1.3)	0.040*
Age 41–64	1.5 (± 1.0)	1.2 (± 0.7)	0.244
Age 65 and above	1.3 (± 1.1)	1.1 (± 0.7)	0.456
Surgical technique, n (%)			
Debridement	77 (95.1%)	65 (100%)	0.129
Number of operations, mean (SD)	3.7 (± 2.8)	4.0 (± 3.7)	0.542
Age 40 and below	2.1 (± 1.5)	4.8 (± 3.3)	0.008*
Age 41–64	4.5 (± 3.1)	3.8 (± 4.6)	0.504
Age 65 and above	3.4 (± 2.5)	3.7 (± 2.9)	0.712
*Post-operative Outcomes*			
Post-operative complications, n (%)	24 (29.6%)	22 (33.8%)	0.596
Medical complications	17 (21.0%)	20 (30.8%)	0.187
Surgical complications	2 (2.5%)	2 (3.1%)	1.000
ICU			
Admission, n (%)	42 (51.9%)	39 (60.0%)	0.402
Age 40 and below	3 (20.0%)	10 (58.8%)	0.036*
Age 41–64	25 (62.5%)	13 (54.2%)	0.602
Age 65 and above	14 (53.8%)	16 (66.7%)	0.399
Mean length of stay [days] (SD)	4.5 (± 7.7)	6.4 (± 10.4)	0.207
Total length of stay in hospital [days], mean (SD)	24.0 (± 25.4)	28.5 (± 30.9)	0.335
Age 40 and below	10.3 (± 9.2)	31.3 (± 35.6)	0.035*
Age 41–64	27.3 (± 29.7)	29.1 (± 38.1)	0.832
Age 65 and above	26.7 (± 22.2)	25.8 (± 17.5)	0.875
Still in hospital after 30 days, n (%)	20 (24.7%)	16 (24.6%)	1.000
Rehabilitation, n (%)	9 (11.1%)	6 (9.2%)	0.789
*Mortality*			
In-hospital mortality, n (%)	11 (13.6%)	10 (15.4%)	0.815
Age 40 and below	0 (0.0%)	1 (5.9%)	1.000
Age 41–64	5 (12.5%)	3 (12.5%)	1.000
Age 65 and above	6 (23.1%)	6 (25.0%)	1.000
30-day mortality, n (%)	1 (1.2%)	0 (0.0%)	1.000
90-day mortality, n (%)	0 (0.0%)	2 (3.1%)	0.197
1-year mortality, n (%)	2 (2.5%)	4 (6.2%)	0.407

The mean age of the total cohort was 56.3 (± 18.4) years old with no significant difference between the study periods (57.4 vs. 55.5 years, *P* = 0.529). Although there were more males overall, there were significantly fewer female patients with NF admitted during the COVID-19 pandemic than the pre-pandemic (21.5% vs. 40.7%, *P* = 0.020). Among 146 patients, 67.8% of them were from Caucasian background. Fifty-five patients (37.7%) were from CALD communities. The proportion of obese patients was 54.8% with an average BMI of 33.6 (± 10.9) kg/m^2^. Approximately two thirds of patients (65.1%) had a pre-existing medical illness and 40.4% of the patients had history of type 2 diabetes mellitus. Half of the patients (50.7%) had a Charlson score of 3 or greater (Table 1).

### Clinical presentation

The mean onset of symptoms till ED presentation of the total cohort was 4.5 (± 3.7) days. The COVID-19 group had a significantly delayed ED presentation of 2.9 days (6.1 vs. 3.2 days, *P* < 0.001). All age groups showed significantly increased mean onset of symptoms till ED presentation during the COVID-19 period compared to the pre-pandemic cohort (Table 1). Swelling, disproportionate pain at the site of injury, and skin erythema were documented in almost all patients (Table 2). The most common sites of injury were perineum (37.0%), lower limb (21.9%), and upper limb (10.3%). Thirty-seven patients of the pre-COVID-19 group (45.7%) and thirty-five patients of the COVID-19 group (53.8%) presented with SIRS (*P* = 0.405). Eight patients of the COVID-19 group had significantly worsening decrease urine output presented compared to the control group (8 vs. 1, *P* = 0.011). There were no statistical differences in all biochemical parameters between the two cohorts (Table 1). The mean LRINEC score was 6.5 with a range of 0–13, and 44.5% of the total cohort were at high risk of NF (LRINEC ≥ 8).

**Table 2:** Patient characteristics and clinical variables

Demographic information	
Age [years], mean (SD)	56.3 (± 18.4)
Range	16–95
Age groups	
Age 40 and below, n (%)	32 (21.9%)
Age 41–64, n (%)	64 (43.8%)
Age 65 and above, n (%)	50 (34.3%)
Gender, n (%)	
Male	99 (67.8%)
Female	47 (32.2%)
Height [cm], mean (SD)	170.2 (± 9.8)
Range	147.0–197.0
Weight [kg], mean (SD)	99.5 (± 37.0)
Range	39.9–248.0
Body mass index (BMI) [kg/m^2^], mean (SD)	33.6 (± 10.9)
Range	17.7–72.5
Obesity, n (%)	69 (54.8%)
Ethnicity, n (%)	
Caucasian	99 (67.8%)
Asian	14 (9.6%)
Middle Eastern	20 (13.7%)
Indigenous	6 (4.1%)
Islander	7 (4.8%)
CALD, n (%)	55 (37.7%)
Smoker, n (%)	47 (32.2%)
*Clinical presentation and symptomology*	
Onset of symptoms till presentation to the Emergency Department [days], mean (SD)	4.5 (± 3.7)
Range	1–28
Age 40 and below	4.4 (± 3.1)
Age 41–64	4.5 (± 4.2)
Age 65 and above	4.5 (± 3.4)
Clinical signs, n (%)	
Hypoaesthesia	12 (8.2%)
Disproportionate pain at the site of injury	142 (97.3%)
Swelling of the skin	138 (94.5%)
Erythema of the skin	136 (93.2%)
Skin crepitus	3 (2.1%)
Haemorrhagic bullae or blisters	12 (8.2%)
Symptoms, n (%)	
Presence of abdominal pain	16 (11.0%)
Site of Injury	
Neck	3 (2.1%)
Chest/Breast	4 (2.7%)
Upper limb	15 (10.3%)
Abdomen	12 (8.2%)
Back	5 (3.4%)
Groin	12 (8.2%)
Buttock	9 (6.2%)
Perineum	54 (37.0%)
Lower limb	32 (21.9%)
Previous trauma to the site	14 (9.6%)
Influenza like symptoms	13 (8.9%)
Decreased urine output	9 (6.2%)
SIRS, n (%)	72 (49.3%)
Sepsis, n (%)	25 (17.1%)
qSOFA, mean (SD)	0.64 (± 0.8)
*Medical comorbidities*	
Pre-existing medical illness, n (%)	95 (65.1%)
Type 1 diabetes mellitus	2 (1.4%)
Type 2 diabetes mellitus	59 (40.4%)
Previous abdominal surgery, n (%)	6 (22.2%)
Charlson comorbidity index, mean (SD)	2.9 (± 2.5)
Charlson score 0, n (%)	30 (20.5%)
Charlson score 1, n (%)	23 (15.8%)
Charlson score 2, n (%)	18 (12.3%)
Charlson score ≥ 3, n (%)	74 (50.7%)
*Biochemical Parameters [normal range], mean (SD)*	
White cell count (× 10^9^/L) [4.0–10.0]	17.5 (± 9.3)
Neutrophil (× 10^9^/L) [2.0–7.0]	14.4 (± 7.4)
C reactive protein (mg/L) [<4.9]	247.6 (± 147.0)
Haemoglobin (g/L) [130–170]	127.3 (± 26.2)
Platelets (× 10^9^/L) [150–400]	265.3 (± 134.6)
Creatinine (µmol/L) [60–110]	151.5 (± 140.3)
Urea (mmol/L) [4.0–9.0]	11.1 (± 9.2)
Albumin (g/L) [33–48]	27.5 (± 7.0)
Lactate (mmol/L) [<1.9]	2.8 (± 2.4)
Sodium (mmol/L) [135–145]	133.5 (± 5.8)
Glucose (mmol/L) [7.8–11.0]	12.5 (± 9.9)
LRINEC category, mean (SD)	6.5 (± 3.3)
Range	0–13
Low risk (≤ 5), n (%)	52 (35.6%)
Moderate risk (6–7), n (%)	29 (19.9%)
High risk (≥ 8), n (%)	65 (44.5%)
*Imaging*	
CT scan, n (%)	76 (52.1%)
*Management*	
ASA status, n (%)	
ASA 1	6 (4.1%)
ASA 2	21 (14.4%)
ASA 3	73 (50.0%)
ASA 4	41 (28.1%)
ASA 5	5 (3.4%)
Time to theatre [hours], mean (SD)	10.9 (± 9.9)
Range	2.8–81.1
Operative time [hours], mean (SD)	1.3 (± 1.0)
Range	0.2–5.6
Surgical technique, n (%)	
Debridement	142 (97.3%)
Others (flap, amputation)	4 (2.7%)
*Post-operative Outcomes*	
Post-operative complications, n (%)	46 (31.5%)
Medical complications	37 (25.3%)
Surgical complications	4 (2.7%)
ICU	
Admission, n (%)	81 (55.5%)
Mean length of stay (SD)	5.3 (± 9.0)
Range	0–62
Total length of stay in hospital [days], mean (SD)	26.0 (± 27.9)
Range	1–154
Still in hospital after 30 days, n (%)	36 (24.7%)
Rehabilitation, n (%)	15 (10.3%)
*Mortality*	
In-hospital mortality, n (%)	21 (14.4%)
Age 40 and below, n (%)	1 (3.1%)
Age 41–64, n (%)	8 (12.5%)
Age 65 and above, n (%)	12 (24.0%)
30-day mortality, n (%)	1 (0.7%)
90-day mortality, n (%)	2 (1.4%)
1-year mortality, n (%)	6 (4.1%)

### Microbiology

At least one identified pathogen was recorded in 132 (90.4%) patients, and 37 (25.3%) patients had more than one type of bacteria found in their wound swabs. The most common pathogens were *Streptococcus milleri* group, *E. coli*, and *Staphylococcus aureus* (Table 3). Patients from the COVID-19 group had significantly higher records of *Klebsiella pneumoniae* (9.2% vs. 1.2%, *P* = 0.045) and *Staphylococcus lugdunensis* (6.2% vs. 0.0%, P = 0.037) than the control group.

**Table 3:** Microbiology results

	Total	Pre-COVID-19 (2017–2019)	COVID-19 (2020–2022)	*P* value
	n = 146	n = 81	n = 65	
Positive wound culture, n (%)	132 (90.4%)	71 (87.7%)	61 (93.8%)	0.264
Polymicrobial infection	37 (25.3%)	18 (22.2%)	19 (29.2%)	0.346
*Streptococcus milleri group*	26 (17.8%)	16 (19.8%)	16 (19.8%)	0.522
*E. coli*	25 (17.1%)	14 (17.3%)	11 (16.9%)	1.000
*Staphylococcus aureus*	18 (12.3%)	9 (11.1%)	9 (13.8%)	0.623
Group A Streptococcus	12 (8.2%)	7 (8.6%)	5 (7.7%)	1.000
MRSA	11 (7.5%)	8 (9.9%)	3 (4.6%)	0.346
Group B Streptococcus	10 (6.8%)	5 (6.2%)	5 (7.7%)	0.752
*Enterococcus faecalis*	7 (4.8%)	2 (2.5%)	5 (7.7%)	0.242
*Klebsiella pneumoniae*	7 (4.8%)	1 (1.2%)	6 (9.2%)	0.045*
*Proteus mirabilis*	4 (2.7%)	1 (1.2%)	3 (4.6%)	0.324
*Staphylococcus lugdunensis*	4 (2.7%)	0 (0.0%)	4 (6.2%)	0.037*
*Pseudomonas aeruginosa*	4 (2.7%)	3 (3.7%)	1 (1.5%)	0.629
*Enterobacter cloacae*	4 (2.7%)	1 (1.2%)	3 (4.6%)	0.324
*Streptococcus dysgalactiae*	3 (2.1%)	1 (1.2%)	2 (3.1%)	0.585
*Enterococcus avium*	2 (1.4%)	0 (0.0%)	2 (3.1%)	0.197
*Proteus vulgaris*	2 (1.4%)	0 (0.0%)	2 (3.1%)	0.197
*Enterococcus faecium*	1 (0.7%)	0 (0.0%)	1 (1.5%)	0.445
Other bacteria	30 (20.5%)	17 (21.0%)	13 (20.0%)	1.000

### Management

The average time from ED admission to theatre of the whole cohort was 10.9 (± 9.9) hours with no significant difference between two groups. Almost all (97.3%) patients had surgical debridement for NF management, and only four patients received other surgical techniques, including three cases for amputation and one case for free flap. Patients aged less than 40 years old had a longer operative time and more operations in the COVID-19 group compared to the control group (1.8 vs. 1.0 h, *P* = 0.040; 4.8 vs. 2.1, *P* = 0.008, Table 1).

### Clinical outcomes

Only two patients of our cohort were positive to COVID-19. The number of patients admitted to ICU during the COVID-19 pandemic was slightly less than before the pandemic (39 vs. 42 patients, *P* = 0.402). There were more patients aged 40 years and below admitted to ICU during the pandemic than the pre-pandemic period (10 vs. 3 patients, *P* = 0.036). The total LoS of patients in this age-group during the COVID-19 pandemic was three times longer than before the pandemic (31.3 vs. 10.3 days, *P* = 0.035). Nevertheless, there was no statistical difference in the total LoS of the COVID-19 group and the control group (28.5 vs. 24.0 days, *P* = 0.335). A total of 46 patients (31.5%) had post-operative complications with no significant differences between two groups (29.6% vs. 33.8%, *P* = 0.596). Of these patients, 21 patients did not survive during their admissions yielding a mortality rate of 14.4%. The numbers of in-hospital mortality of the COVID-19 group and the control group were 10 and 11, respectively (*P* = 0.815, Table 1). All patients with positive COVID-19 in our study survived.

### Impacts of lockdown periods

**Fig. 2:**
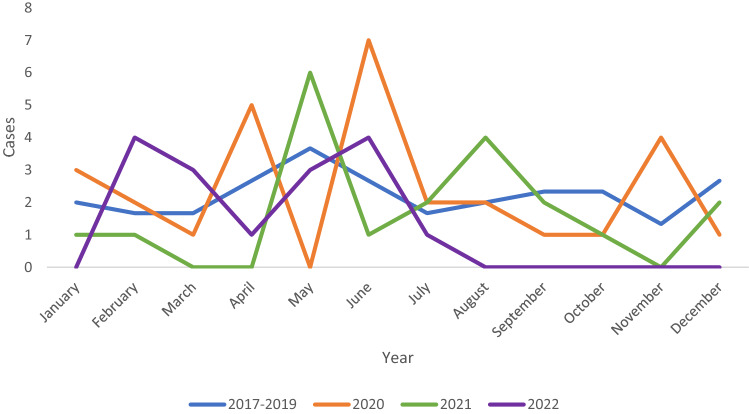
Number of necrotising fasciitis cases in a month from January 2017 to October 2022 (First lockdown period March–July 2020; Second lockdown period August–October 2021)

The number of NF cases slightly increased during the first lockdown period (15 vs. 12, *P* = 0.853, Fig. 2). Patients with NF presented slightly later (4.8 vs. 3.2 days, *P* = 0.233), however, the proportion of septic patients was higher (20.0% vs. 5.0%, *P* = 0.119) with a significantly prolonged operative time (1.5 vs. 1.0, *P* = 0.028) and longer mean LoS in ICU (6.6 vs. 2.8, *P* = 0.021). The 1-year mortality rate of COVID-19 group was higher than the control group (20.0% vs. 2.5%, *P* = 0.057), but there were no differences in in-hospital, 30-day, and 90-day mortality rates between two groups.

During the second lockdown period, patients of the COVID-19 group presented to ED significantly later than the control group (8.0 vs. 2.9 days, *P* = 0.001). The number patients with NF during the second lockdown period was slightly less than previous years (7 vs. 6, *P* = 0.232, Fig. 2). Nevertheless, there were no statistical differences in the clinical outcomes between two groups.

## Discussion

Imposed social restrictions and lockdown during the COVID-19 pandemic have largely resulted in delayed presentations and worse clinical outcomes of patients with NF. The COVID-19 group had a significantly prolonged mean onset of symptoms prior to hospital admission of 6.1 days, whereas a previous systemic review and meta-analysis reported the mean time of 4.5 days (range 1.0 to 13.3 days) [13]. Our finding was congruent with our previous study showing a decrease in ED presentations and an average delayed presentation of 4.1 days of NF patients during the pandemic [6, 8]. A Spanish study of general surgery reported the same pattern with an increased delay of almost 24 h from the onset of symptoms to arrival at an ED and a decrease in daily cases of emergency general surgeries [15]. Nevertheless, this was contrary to the findings of McGee, et al. [16], which showed a 113% increase in cases of NF during the COVID-19 pandemic in the United States. One of the reasons for delayed presentations was patients considered respiratory symptoms of COVID-19 more serious than other non-specific symptoms [17]. Australia was one of the harshest countries to impose tough regulations and punishments during the pandemic and we believe that those law enforcements indirectly created a physical barrier to accessing healthcare services [17]. Nevertheless, health promotion programs should be implemented to educate people on the crucial role of early health-seeking behaviour for life-threatening conditions, which can still occur during a global pandemic.

The progression of signs and symptoms of NF can be vague and often neglected with the literature reporting rates from 41.0% to 96.0% [18]. Common symptoms of early NF include erythema (72%), pain (72%), and swelling (75%) [11, 18]. We found almost all patients presented with swelling, pain, and erythema in our study (Table 2). These findings are similar to soft tissue infections making early diagnosis sometimes challenging. The SIRS response was present in both groups with no significant difference (Table 1), however, it is not unusual for fever, tachycardia, and cutaneous manifestations to be initially absent [11, 19]. A retrospective review of 89 patients reported that only 53% were febrile and 18% were hypotensive at presentation [20]. Similarly, there were less than 50% of patients in our study who met the criteria for SIRS, but only 17.1% of them met the criteria of sepsis. Skin crepitus, haemorrhagic bullae, blisters, and sensory and motor deficits are very late signs of NF [20, 21]. Therefore, the surgical hallmark for consideration of NF is still disproportionate pain in a superficial soft tissue infection despite limited data on sensitivity and specificity for the diagnosis [19]. Apart from oliguria, the clinical symptoms or signs of NF found between two groups were not significantly different (Table 1).

The LRINEC score has been used to assist clinicians to assess the risk of NF in those who presented with soft tissue infection based on its adjunct biochemical scoring system [14]. A LRINEC score of 6 or greater is considered to be a strong indicator of NF (93% sensitive, 94% specific), but it must be considered in the context of the overall clinical presentation [14]. The patients in our study had the average LRINEC score of 6.5 (range between 0 and 13), which was equivalent to the mean score of 6.06 in a previous systematic review [22]. Although the LRINEC scores of the two groups were not significantly different (6.5 vs. 6.4), we found that patients who did not survive had a mean LRINEC score of 7.4 ranging from 2 to 13. This is consistent with a retrospective analysis, reporting significantly higher mortality rate in patients with LRINEC score ≥ 6 [23].

The proportion of positive wound culture in our study (90.4%) was higher than previous studies ranging from 40.3% to 77.0% [24, 25]. Like others, we also found that monomicrobial infection was more prevalent [25, 26]. We had expected a higher proportion of polymicrobial infections in the context of considerable proportion of elderly patients aged 65 years and above (34.3%) and high percentage of patients with pre-existing medical illness (65.1%) [11].

*Following the Streptococcus milleri group of bacteria (S. anginosus, S. constellatus and S. intermedius), E. coli* was the most common single pathogen identified in our study (17.1%), and it was more prevalent than staphylococci and most streptococci isolated in other studies [24]. This was in line with an increased incidence of *E. coli* isolated from the wound of patients with NF in a longitudinal study of Bodansky, et al. [24].

NF can be managed with antibiotic therapy, haemodynamic support, and prompt surgical exploration and debridement of necrotic tissue [19]. The mortality rate can be increased by nine fold with a delay of debridement more than 24 h [14, 27]. Nawjin, et al. reported that better mortality rate was achieved when surgical debridement was done promptly within 12 h since the initial presentation (OR = 0.41) in a systematic review and meta-analysis [13]. In our study, 71.9% of patients underwent surgeries in less than 12 h from their admissions but having surgeries more than 12 h (OR = 1.029, 95% CI 0.369–2.864) and 24 h (OR = 1.539, 95% CI 0.304–7.807) after presentation was surprisingly not associated with increased mortality rate. The average time to operation after presentation was 10.9 h without delay between two cohorts, which was substantially shorter than the mean time to operating theatre of 16.2 h of Quah, et al. [28]. This was contrary with the findings of Dick, et al. [29] which showed an increase in the mean operative time of emergency surgery during the COVID-19 pandemic. During the first lockdown, our findings showed the mean operative time increased compared with to the previous non-COVID years (1.5 vs. 1.0 h, *P* = 0.028). Similarly, patients of the age group 40 years and below underwent longer surgeries during the pandemic era (1.8 vs. 1.0 h, *P* = 0.040). Matsuyama, et al. reported a correlation between higher mortality and morbidity rates and duration of operations, which was longer than 2 h in their retrospective study of 562 patients [30]. Prolonged operative time was found to be a result of multiple factors including COVID-19 tests beforehand and additional steps for anaesthetic and operative setup during the pandemic. Other factors that may have contributed a longer operative time included time taken for donning PPE during aerosol-generating procedures in the COVID-19 pandemic [31]. During this global pandemic, operative and anaesthetic times were found to be longer for trauma surgeries and caesarean sections in the studies of Khadabadi, et al. [32] and Cuerva, et al. [33], respectively.

The in-hospital mortality rates of the COVID-19 and the non-COVID-19 groups were 15.4% and 13.6%, respectively, keeping within the range of mortality rate of pre-COVID-19 studies (5.8% to 25.8%) [20, 28, 34, 35]. However, we reported patients aged less than 40 years old had greater number of operations (4.8 vs. 2.1, *P* = 0.008), higher number of ICU admission (10 vs. 3, *P* = 0.036), and longer total LoS (31.3 vs. 10.3 days, *P* = 0.035) compared to their counterparts. These results could be attributed to their significant delayed presentation of 3.3 days (5.9 vs. 2.6 days, *P* = 0.001), which was even longer than the overall delayed presentation of 2.9 days (Table 1). Kobayashi, et al.’s retrospective study of 47 patients reported that an increased number of surgical debridement was associated with delayed treatment for NF [36].

This study, to our knowledge, is the first one examining the presentation and clinical outcomes of NF in Australia after more than two years following the announcement of the global coronavirus pandemic. The retrospective design is the main factor contributing to limitations of our study because of its potential selection bias and all cases may have not been included. Surveillance of the disease and data-informed decision-making are key to achieve good service and high standard of patient care.

## Conclusion

The delayed presentations of NF during the COVID-19 pandemic were likely attributed to the extended periods of lockdown, the severe penalties if found uncompliant, and changes in health seeking behaviours. Patients, who were 40 years old and younger, were most likely to delay their presentations to ED and to experience prolonged operative time with higher number of operations and greater LoS. Despite late presentations, satisfactory clinical outcomes for NF cases were achieved without any significant differences in post-operative complication and mortality rates. People should be encouraged and empowered to seek healthcare services early for life-threatening conditions via health promotion programs.

## Footnotes

1 Bankstown-Lidcombe Hospital, Liverpool Hospital, Campbelltown Hospital, Fairfield Hospital.
